# Integrative Bioinformatics Approaches for Identification of Drug Targets in Hypertension

**DOI:** 10.3389/fcvm.2018.00025

**Published:** 2018-04-04

**Authors:** Daiane Hemerich, Jessica van Setten, Vinicius Tragante, Folkert W. Asselbergs

**Affiliations:** ^1^Department of Cardiology, University Medical Center Utrecht, University of Utrecht, Utrecht, Netherlands; ^2^CAPES Foundation, Ministry of Education of Brazil, Brasília, Brazil; ^3^Durrer Center for Cardiovascular Research, Netherlands Heart Institute, Utrecht, Netherlands; ^4^Institute of Cardiovascular Science, Faculty of Population Health Sciences, University College London, London, United Kingdom; ^5^Farr Institute of Health Informatics Research and Institute of Health Informatics, University College London, London, United Kingdom

**Keywords:** hypertension, blood pressure, epigenetic regulation, GWAS, data integration, functional annotation, drug target identification.

## Abstract

High blood pressure or hypertension is an established risk factor for a myriad of cardiovascular diseases. Genome-wide association studies have successfully found over nine hundred loci that contribute to blood pressure. However, the mechanisms through which these loci contribute to disease are still relatively undetermined as less than 10% of hypertension-associated variants are located in coding regions. Phenotypic cell-type specificity analyses and expression quantitative trait loci show predominant vascular and cardiac tissue involvement for blood pressure-associated variants. Maps of chromosomal conformation and expression quantitative trait loci (eQTL) in critical tissues identified 2,424 genes interacting with blood pressure-associated loci, of which 517 are druggable. Integrating genome, regulome and transcriptome information in relevant cell-types could help to functionally annotate blood pressure associated loci and identify drug targets.

## Introduction

Elevated blood pressure (BP) or hypertension is a heritable chronic disorder (1–3), considered the single largest contributing risk factor in disease burden and premature mortality (4). High systolic and/or diastolic BP reflects a higher risk of cardiovascular diseases (4). Genome-wide association studies (GWAS) have found association of 905 loci to BP traits (systolic - SBP, diastolic - DBP and pulse pressure -PP) to date (Table S1) (5–33). The use of larger sample sizes has helped to identify additional variants, as demonstrated by the most recent study including over 1 million people that has identified 535 novel BP loci (33). Still, this collective effort thus far has not entirely elucidated the complete genetic contribution to BP, estimated to be approximately 50–60% (34).

**Figure 1:**
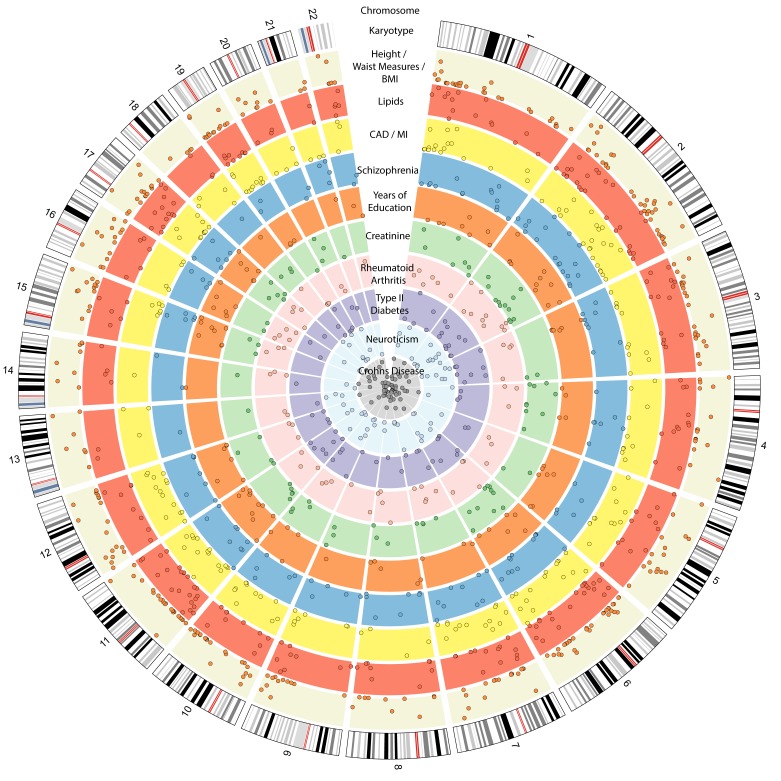
Circos plot showing the 10 traits from the GWAS catalog (37) with the largest number of loci also associated to BP, as identified by PhenoScanner (38) at *p* < 0.05 (Supplemental Methods). The outer ring represents the genomic/chromosomal location (hg19). The following inner rings show the associations to different traits. Beige: body measurements (height, body mass index (BMI), weight, waist/hip ratio, hip circumference, waist circumference. *N* = 358). Red: lipids (high-density lipoprotein (HDL), low-density lipoprotein (LDL), triglycerides, total cholesterol. *N* = 226). Yellow: coronary artery disease (CAD)/myocardial infarction (MI) (*N* = 206). Blue: schizophrenia (*N* = 135). Orange: years of education attendance (*N* = 101). Light green: creatinine (*N* = 88). Light pink: rheumatoid arthritis (*N* = 78). Purple: type II diabetes (*N* = 73). Light turquoise: neuroticism (*N* = 69). Light grey: Crohn’s disease (*N* = 67).

To add to this complexity, 90.7% of the 905 BP-associated index variants are located in intronic or intergenic regions (Table S1). Causal variants are also difficult to pinpoint because of linkage disequilibrium (LD) (35). There is now vast evidence that non-coding variants associated with disease interrupt the action of regulatory elements crucial in relevant tissues for that particular disease (36). BP loci are not only linked to cardiovascular disease but also to other diseases (Figure 1), suggesting that BP-associated variants can result in a wide range of phenotypes. Tissue specificity of genetic loci may be relevant for organ specific disease progression. For example, variants altering expression in heart may more likely affect disease progression through heart-mediated processes rather than kidney-mediated processes, and some patients may suffer of left ventricular hypertrophy while others may develop nephropathy. Thus, investigating the influence of BP variants in critical cell-types is essential in understanding disease risk and biology, and assessing the possible translation of an associated locus into a drug target. The public availability of regulatory annotations in several tissues by projects such as ENCODE (39), Roadmap (40) and GTEx (41, 42) has enabled integration of epigenetic modifications, expression quantitative trait loci (eQTLs) and –omics information with GWAS data. Integrative approaches are useful for prioritizing genes from known GWAS loci for functional follow-up, detecting novel gene-trait associations, inferring the directions of associations, and potential druggability (43–46).

**Figure 2:**
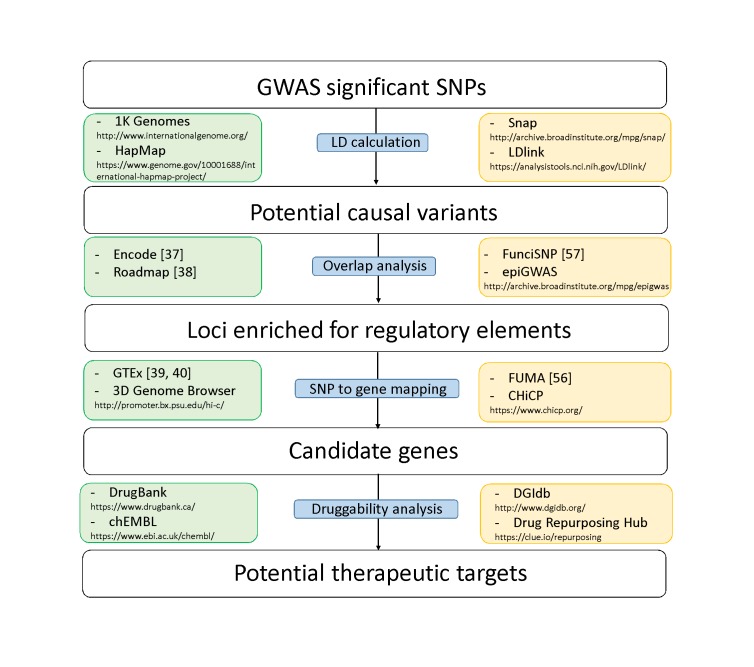
Diagram of analytical steps that can be followed for variant prioritization and translation of association to a potential drug target. Each step is accompanied by examples of publicly available data (green boxes on the left) and tools (yellow boxes on the right) that can be used.

Here we summarize the advances made in recent years towards unraveling the mechanisms of non-coding BP variants in disease progression with the resources mentioned above. We focus on integrative approaches that aim to prioritize BP-associated SNPs located in regulatory regions of the genome for follow-up studies (Figure 2). Genetic and molecular aspects of hypertension have been reviewed previously by others (47, 48).

## Integrative Approaches Using –Omics Data

Remarkable advances have been made recently towards a better comprehension of BP genetics, the biology of disease and translation towards new therapeutics, boosted by the widespread application of high-throughput genotyping technologies. At the same time, most BP-associated variants are non-coding, making the conversion of statistical associations into target genes a great challenge. SIFT (49, 50), PROVEAN (51), PolyPhen (52), CONDEL (53) and more recently CADD (54) are scoring algorithms developed for predicting the effect of amino acid changes. Only 98 out of the 905 lead BP-associated SNPs reflect a CADD score above 12.37 (Table S2), a threshold suggested by Kicher et al. as deleterious (54). However, the causal variant inside the locus might reflect a different CADD score than the lead SNP, and pinpointing the mechanisms disturbed by the variation remains a challenge. 

New strategies that make use of regulatory annotations in disease-relevant tissues have greatly expanded our ability to investigate the processes involved in BP. In particular, annotation of histone modifications and regions of open chromatin allow the identification of active transcription in specific-cell types. Similarly, maps of DNA variants affecting expression in a cell-type specific manner will be integral in BP loci interpretation. A list of cardiovascular-related cell-types researched by the ENCODE Project is presented by Munroe et al. (55). Such data can be integrated with GWAS results using bioinformatics tools (56–58). For instance, FUMA provides extensive functional annotation for all SNPs in associated loci and annotates the identified genes in biological context (57). FunciSNP investigates functional SNPs in regulatory regions of interest (58). Ensemble's Variant Effect Predictor (VEP) determines the effect of variants on genes, transcripts, and protein sequence, as well as regulatory regions, also outputting SIFT, Polyphen and CADD scores for each variant, among other information (59). Although such integrative tools are useful for variant prioritization and interpretation, not all take into consideration tissue specificity aspects. RegulomeDB, for example, is a database that annotates SNPs with known and predicted regulatory elements in the intergenic regions of the human genome, calculating a score that reflects its evidence for regulatory potential (60). However, the scoring procedure can only be performed across all available tissue types. In addition, several databases containing a broad range of tissues were made publicly available since the last update of RegulomeDB, that could be included in the tool. Together, these resources have been useful in prioritizing genes and variants in associated loci for functional follow-up experiments in many post-GWAS analyses, and can be implemented in interpretation of BP-associated loci.

### Transcription Regulation: Histone Modifications and Open Chromatin

**Figure 3:**
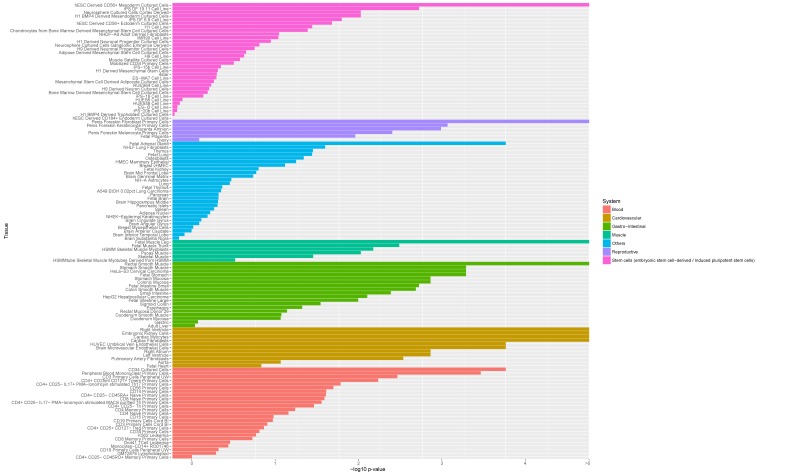
Ranked tissues after phenotypic cell-type specificity analysis of 905 BP SNPs using 125 H3K4me3 datasets on human tissue (Supplemental Methods, Table S3).

As genomic coordinates of active regulatory elements may be mapped using unique functions of chromatin, the characterization of chromatin changes in the genome in specific cell-types can be used to identify DNA variants disturbing active regulatory elements. The four core chromatin histones, H2A, H2B, H3 and H4, can suffer posttranslational modifications, such as acetylation or methylation (61). These histone modifications indicate active (euchromatin) or repressed (heterochromatin) chromatin structure, defining regulation and gene transcription (62, 63). Acetylation of histones H3 and H4, and H3 methylation at Lys4 (H3K4me3), for instance, correlate with gene transcription, whereas methylation at Lys9 correlates with gene silencing (62, 64). These modifications provide a robust readout of active regulatory positions in the genome, and have been employed for annotation in several studies (23). Histone modifications influencing arterial pressure have been observed in many tissues, including vascular smooth muscle (65). An updated phenotypic cell-type specificity analysis of the 905 BP loci using H3K4me3 mark in 125 tissues is shown in Figure 3. The most significant cell-types are cardiovascular-related (Supplemental Methods, Table S3). Other tissues with high rank in specificity are smooth muscle, fetal adrenal gland, embryonic kidney cells, CD34 and stem-cell derived CD56 +mesoderm cultured cells. These results are consistent with analyses using DNase I hypersensitivity sites (DHSs), which indicate likely binding sites of transcription factors. These results add more evidence that BP loci are enriched on regions of open chromatin (19, 20, 23, 33) (Figure S1), regulating transcription in a broad range of tissues.

### Methylation

In addition to histone modifications that promote transcription, BP loci have also been studied for their enrichment on DNA methylation, known to have the opposite regulatory effect. The methylation of CpG sites, presented by CpG islands in promoters, affects binding of transcription factors, resulting in gene silencing (66, 67). Abnormal CpG methylation is found in hypertension (68–70), and in many other complex diseases (71, 72). Recently, Kato et al. identified a ~2 fold enrichment associating BP variants and local DNA methylation (19). The study also demonstrates that DNA methylation in blood correlates with methylation in several other tissues. These observations add to previous indications on the function of DNA methylation in regulating BP.

### Measuring the Impact of BP Risk Alleles on Gene Expression: eQTLs

Expression quantitative trait loci (eQTL) are regions harbouring nucleotides correlating with alterations in gene expression (73). Linking transcription levels to complex traits has been a follow-up step adopted by many studies (43, 74–76), driven by the increase in available data of expression patterns across tissues and populations (33, 46, 77–81). Warren et al. found that 55.1% of their identified BP-associated loci have SNPs with eQTLs in at least one tissue from GTex repository (41), with arterial tissue most frequently observed (29.9% of loci had eQTL in aorta and/or tibial artery) (21). A great enrichment of eQTLs in artery was also observed by Evangelou et al., who identified 92 novel loci with eQTL enrichment in arterial tissue and 48 in adrenal tissue (33). In summary, these studies also suggest that BP loci exert a regulatory effect mostly in vascular and cardiac tissues.

### Finding the Targets: Chromosome Confirmation Capture Techniques

Mapping variation to target genes is one of the greatest challenges in the post-GWAS era, and different strategies have been developed to this end (82). One approach is the use of chromosome confirmation capture [3C (83), 4C (84, 85), Hi-C (86, 87)]. These techniques capture chromosome interactions (88), resulting in networks of interacting genetic loci (84, 85).

Warren et al. made use of this resource to investigate the target genes of non-coding SNPs, using Hi-C data from endothelial cells (HUVECs). Distal potential genes were found on 21 loci, and these genes were enriched for regulators of cardiac hypertrophy in pathway analysis (20). Kraja et al. also explored long-range chromatin interactions using endothelial precursor cell Hi-C data (89, 90), finding the link between an associated loci and a gene known to affect cell growth and death (91). More recently, Evangelou et al. used chromatin interaction Hi-C data from HUVECs (92), neural progenitor cells (NPC), mesenchymal stem cells (MSC) and tissue from the aorta and adrenal gland (93) to identify distal affected genes. They found 498 novel loci that contained a potential regulatory SNP, and in 484 loci long-range interactions were found in at least one cell-type (33).

**Figure 4:**
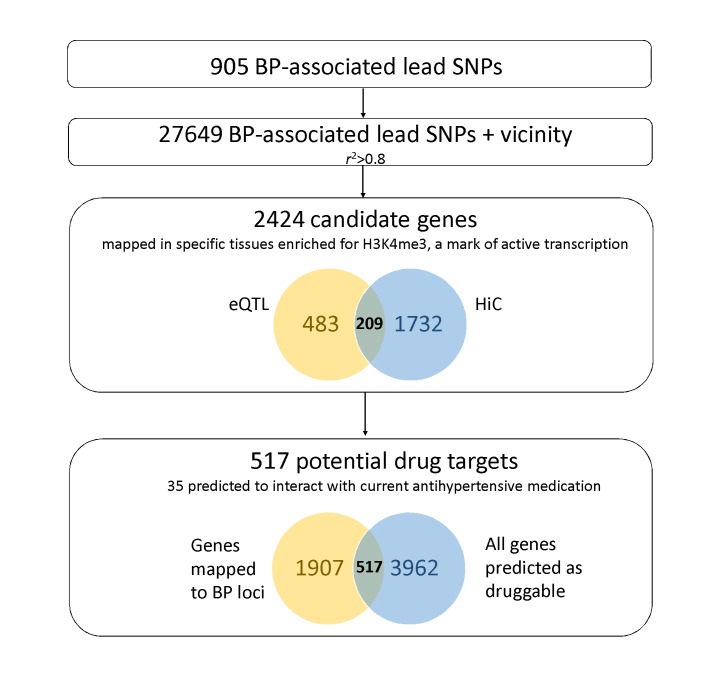
Diagram illustrating the results of our integrative approach.

A list of human HiC data available on BP relevant tissues is presented in Table S4. An updated version of variant to gene mapping making use of this chromatin conformation data is shown in Table S5. Promoter regions of 1,941 genes were found to interact with the 27,649 candidate SNPs (905 BP associated SNPs and vicinity) (Supplemental Methods, Figure 4). Integration with eQTL data on relevant tissues confirmed 209 of the genes mapped, and added additional 483 genes. One main goal of understanding biological mechanisms of GWAS associations and affected genes is to be able to therapeutically target them. Assessment of the druggability of a BP-associated locus depends on several factors, but overlap of these results with a recent effort on druggability suggests that 517 of these 2,424 genes are druggable (94), and 35 mapped genes are also predicted to interact with common drugs for treatment of hypertension (Table S2, Figure 4, Supplementary Methods). Interestingly, 1,774 of the genes mapped are physically located outside BP-associated loci. These results support the hypothesis that BP GWAS loci act on tissue specific regulatory gene networks. Importantly, they also show that the use of long range chromatin interaction maps can reliably identify target genes even outside the risk locus.

## Discussion and Conclusions

GWAS have pinpointed over 900 loci associated with BP, and increasing sample size has shown to be crucial to identify more signals (33). However, efforts are needed to translate these results into biological inferences on causal mechanisms and understanding of disease biology. The integration of data beyond the DNA sequence is crucial to identify genes involved in BP regulated by epigenetic mechanisms.

BP variants show eQTL, histone modification and open chromatin enrichment in a broad range of tissues, mostly vascular and cardiac-related. As the interplay of regulatory elements is highly cell-type specific, the study of changes that influence chromatin structure and accessibility needs to be extended to a broad range of tissues and conditions, including disease and its stages. Rosa-Garrido et al. observed chromatin structural abnormalities when comparing healthy and diseased cardiac myocytes, concluding that heart failure involves altered enhancer-gene interactions (95). Thus, alterations in chromatin structure underlying heart disease perturbs significant interactions that contribute to gene expression. This finding suggests that high resolution chromatin conformation and epigenetic data in disease state can help in understanding how regulatory variants confer risk to disease. The availability of data in different populations will also allow fine-mapping and functional annotation across ethnic groups.

By mapping of BP-associated variants to genes using maps of chromosomal conformation in specific cell-types, we identified 1,941 genes, of which 209 show supported by eQTL mapping. Of all genes mapped (*n* = 2,424), 517 are predicted as druggable and 35 are predicted to interact with common antihypertensive drugs. These include successful cases such as *APOB* gene, predicted to be targeted by Ibersartan, an angiotensin II receptor antagonist used mainly for the treatment of hypertension (96). Interestingly, in this analysis we were also able to identify *ABCC9* gene on both eQTL and HiC mapping, a gene that interacts with Minoxidil. Although originally developed as an antihypertensive vasodilator, side effects provided limitations and currently its main application occurs topically for treatment of hair loss (97, 98). This highlights the several factors involved in druggability of a target and need for extensive validation and trials. With *in-silico* experimental evidence supporting a plausible mechanism for association, definitive assignment of functions to putative cis-regulatory elements requires perturbation of these elements. Although the majority of associated variants add only modest effects on risk, more studies suggest combinations of SNPs are frequently necessary in order to explain these effects (99–101). CRISPR–Cas9 (Clustered Regularly Interspaced Short Palindromic Repeats) editing technology (102) permits targeted manipulation of epigenetic mechanisms linked to risk alleles (103). Finally, genes that show consequent differential expression can be further validated *in vivo* with the use of animal models.

In summary, the integrative approaches presented in this review help understanding the underlying biology of GWAS loci by mapping SNPs to genes and determine cell and tissue-specificity. The increase in availability of regulatory data in a broad range of tissues and disease states will expand the possibilities for integration and interpretation of association results. Studies validating the genes prioritized may identify new drug targets, enabling more effective prevention and treatment of hypertension and its consequences.

## Author Contributions

DH, VT and FA contributed in study conception and design. DH was responsible for analysis and interpretation of data and drafting of manuscript. DH, VT, JS and FA provided critical revision and final approval of the manuscript.

## Conflict of Interest Statement

The authors declare that the research was conducted in the absence of any commercial or financial relationships that could be construed as a potential conflict of interest.
