# Supplementary material for: Integrative Bioinformatics Approaches for Identification of Drug Targets in Hypertension
Source: Front Cardiovasc Med. 2018 Apr 4;5:25. doi: 10.3389/fcvm.2018.00025 (PMC5894467; doi:10.3389/fcvm.2018.00025)
Supplement: Supplementary file 4 [file Table4.docx]

**Supplemental Table 4.** Tools for integration and visualization of publically available datasets of chromosomal conformation experiments performed on tissues relevant for BP.

| Tool | URL | Adrenal Gland | Aorta | Endothelial Precursor Cell | HUVEC | LV | RV |
| --- | --- | --- | --- | --- | --- | --- | --- |
| 3D Genome Browser | http://promoter.bx.psu.edu/hi-c/ |  |  |  | x | x | x |
| Capture HiC Plotter (CHiCP) | https://www.chicp.org/ |  |  | x |  |  |  |
| FUMA | http://fuma.ctglab.nl/ | x | x |  |  | x | x |
