# Supplementary material for: Integrative Bioinformatics Approaches for Identification of Drug Targets in Hypertension
Source: Front Cardiovasc Med. 2018 Apr 4;5:25. doi: 10.3389/fcvm.2018.00025 (PMC5894467; doi:10.3389/fcvm.2018.00025)
Supplement: Supplementary file 5 [file DataSheet1.docx]

Supplementary Material

Integrative bioinformatics approaches for identification of drug targets in hypertension

Daiane Hemerich*, Jessica van Setten, Vinicius Tragante, Folkert W. Asselbergs

*** Correspondence:** Corresponding Author: D.Hemerich@umcutrecht.nl

# Supplemental Methods

**1.1 Association lookups with other diseases**

We used PhenoScanner [1] database to evaluate cross-trait effects for the 905 blood pressure (BP) -associated sentinel SNPs including proxies in linkage disequilibrium (LD, *r^2^* ≥ 0.8) with variants of the GWAS catalog [2] at *p* < 0.05.

**1.2 Prediction of coding consequences**

We used Variant Effect Predictor (VEP) from Ensemble [3] to predict the coding consequences of the canonical transcript of each lead BP-associated SNP, as well as its CADD score [4]. Predicted coding consequences other than "missense_variant" were renamed as "synonymous_variant".

**1.3 Phenotypic cell-type specificity**

We obtained publicly available data on H3K4me3 histone modification on 126 tissues from ENCODE [5] and NIH Roadmap Epigenomics Mapping Consortium [6] (Supplemental Table 1). We downloaded alignment files in hg-19 coordinates of both treatment and control. Duplicated, unmapped reads and reads with mapping quality less than 5 (-b -F 4 -q 5) were removed using Samtools v1.3 [7]. Peak calling was performed using MACS v2.1.0 [8] using the respective input samples, -gsize=hg -nomodel parameters, and estimated fragment sizes (-extsize) predicted by PhantomPeakQualTools v1.1 [9]. We used the method described by Trynka *et al.* [10] for phenotypic cell-type specificity analysis, in which a locus is defined by identifying SNPs in tight LD with each variant (*r^2^* ≥ 0.8), using data from the 1000 Genomes Project [47]. Each variant is scored based on the distance and height of the nearest peak. If the physical distance to the nearest peak is more than 2.5 kb, then the score is set to 0 to obviate any confounding distal effects. The statistical significance of cell-type specificity is assessed by deriving a 95^th^ percentile threshold based on the permutation of 10,000 matched sets of SNPs not associated with the phenotype.

We used FORGE [11] to investigate cell-type-specific enrichment (FDR, *p* < 0.05) within DNase I–hypersensitive sites in 125 cell samples from ENCODE project [5], using default parameters. Briefly, FORGE compares the frequency of query variants in different cell types with a reference set of 1204 control variants from the GWAS catalog [2] with discovery *p* < 5 × 10^−8^ in European ancestry populations. For each cell-type and *p*-value threshold, the enrichment of query variants mapping to footprints is expressed as a *p*-value derived from a logistic mixed effect model.

**1.4 Variant to gene mapping through chromosomal conformation**

We used FUMA v1.3.0 [12] to map the 905 BP-associated sentinel SNPs and proxies in LD (*r^2^* ≥ 0.8) to genes in relevant cell-types. We used maps of chromosomal conformation (HiC) in HUVEC [13], adrenal gland, aorta, left ventricle, right ventricle [14] and eQTL from GTEx v7 in adrenal gland, aorta, artery coronary, artery tibial, atrial appendage and left ventricle [15], keeping default parameters.

**1.5 Interaction of mapped genes and antihypertensive drug targets**

In order to assess the overlap between drug targets and BP loci, we obtained a list of medications used to treat hypertension at WebMD [16] and checked for their predicted interactions with gene products using DGIdb [17, 18]. We used the genes identified by our integrative mapping as representatives of each significant SNP to find matches with the output of DGIdb. A list of genes predicted as druggable was retrieved from the study by Finan *et al.* (2017) [19].

# Supplemental Figures and Tables

## Supplemental Figures


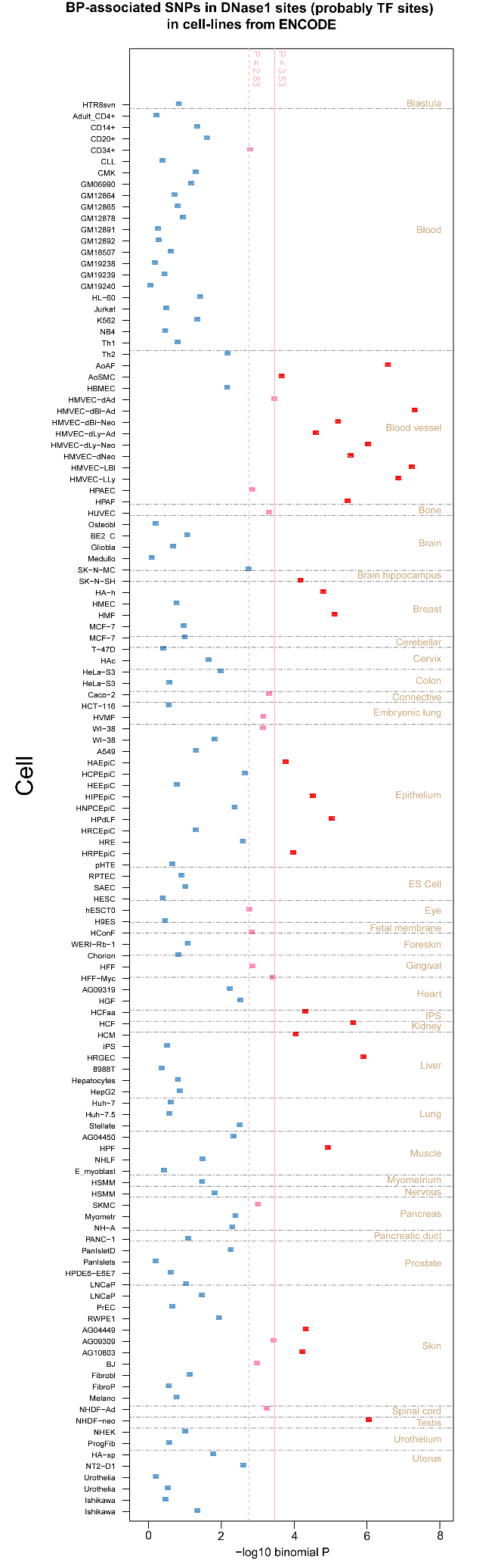


**Supplemental Figure 1.** Phenotypic cell-type specificity analysis results using DNase 1, showing enrichment predominantly on cardiovascular tissues.

## Supplemental Tables

**Supplemental Table 1.** Summary information on the 905 BP-associated variants identified to date.

**Supplemental Table 2.** Results of the integrative approach. CADD score and coding consequences predicted for the canonical transcript of each lead BP-associated SNP. “Gene HiC” and “Gene eQTL” columns show genes mapped by HiC and/or eQTL in relevant tissues. Genes identified by both resources are highlighted in red. Column "Gene predicted as druggable" shows which mapped gene is predicted to be druggable according to Finan *et al.* (2017). Last column shows genes predicted to interact with current antihypertensive medicines.

**Supplemental Table 3.** Phenotypic cell-type specificity analysis results using H3K4me3, showing enrichment predominantly on cardiovascular tissues.

**Supplemental Table 4.** Tools for integration and visualization of publically available datasets of chromosomal conformation experiments performed on tissues relevant for BP.

**Supplemental Table 5.** Protein-coding genes mapped to the 905 BP-associated loci using maps of chromosomal conformation (HiC) in relevant cell-types (HUVEC, adrenal gland, aorta, endothelial precursor cells, LV=left ventricle, RV=right ventricle) and eQTL (in adrenal gland, aorta, artery coronary, artery tibial, atrial appendage, LV). Positions given in GRCh37 (hg19) genomic coordinates.

**References**

1. Staley, J.R., et al., *PhenoScanner: a database of human genotype-phenotype associations.* Bioinformatics, 2016. **32**(20): p. 3207-3209.

2. Hindorff, L.A., et al., *Potential etiologic and functional implications of genome-wide association loci for human diseases and traits.* Proc Natl Acad Sci U S A, 2009. **106**(23): p. 9362-7.

3. McLaren, W., et al., *The Ensembl Variant Effect Predictor.* Genome Biol, 2016. **17**(1): p. 122.

4. Kircher, M., et al., *A general framework for estimating the relative pathogenicity of human genetic variants.* Nat Genet, 2014. **46**(3): p. 310-5.

5. Consortium, E.P., *An integrated encyclopedia of DNA elements in the human genome.* Nature, 2012. **489**(7414): p. 57-74.

6. Roadmap Epigenomics, C., et al., *Integrative analysis of 111 reference human epigenomes.* Nature, 2015. **518**(7539): p. 317-30.

7. Li, H., et al., *The Sequence Alignment/Map format and SAMtools.* Bioinformatics, 2009. **25**(16): p. 2078-9.

8. Feng, J., T. Liu, and Y. Zhang, *Using MACS to identify peaks from ChIP-Seq data.* Curr Protoc Bioinformatics, 2011. **Chapter 2**: p. Unit 2 14.

9. Kharchenko, P.V., M.Y. Tolstorukov, and P.J. Park, *Design and analysis of ChIP-seq experiments for DNA-binding proteins.* Nat Biotechnol, 2008. **26**(12): p. 1351-9.

10. Trynka, G., et al., *Chromatin marks identify critical cell types for fine mapping complex trait variants.* Nat Genet, 2013. **45**(2): p. 124-30.

11. Dunham, I., et al., *FORGE : A tool to discover cell specific enrichments of GWAS associated SNPs in regulatory regions.* bioRxiv, 2014.

12. Watanabe, K., et al., *Functional mapping and annotation of genetic associations with FUMA.* Nat Commun, 2017. **8**(1): p. 1826.

13. Rao, S.S., et al., *A 3D map of the human genome at kilobase resolution reveals principles of chromatin looping.* Cell, 2014. **159**(7): p. 1665-80.

14. Schmitt, A.D., et al., *A Compendium of Chromatin Contact Maps Reveals Spatially Active Regions in the Human Genome.* Cell Rep, 2016. **17**(8): p. 2042-2059.

15. Consortium, G.T., et al., *Genetic effects on gene expression across human tissues.* Nature, 2017. **550**(7675): p. 204-213.

16. WebMD. *Drugs & Medications Search*. 2018. Accessed on: 20/02/2018; Available from: <https://www.webmd.com/drugs/condition-1432-High%20Blood%20Pressure%20%20Hypertension%20.aspxdiseaseid=1432&diseasename=High+Blood+Pressure+(Hypertension>).

17. Wagner, A.H., et al., *DGIdb 2.0: mining clinically relevant drug-gene interactions.* Nucleic Acids Res, 2016. **44**(D1): p. D1036-44.

18. Griffith, M., et al., *DGIdb: mining the druggable genome.* Nat Methods, 2013. **10**(12): p. 1209-10.

19. Finan, C., et al., *The druggable genome and support for target identification and validation in drug development.* Sci Transl Med, 2017. **9**(383).
